# Supplementary material for: Hybrid Models and Biological Model Reduction with PyDSTool
Source: PLoS Comput Biol. 2012 Aug 9;8(8):e1002628. doi: 10.1371/journal.pcbi.1002628 (PMC3415397; doi:10.1371/journal.pcbi.1002628)
Supplement: Text S4 — Complete source code for the PyDSTool package (version 0.88.120504). Includes API documentation and help files linking to web pages. This file is identical to the current public release on Sourceforge.net. (ZIP) [file pcbi.1002628.s004.zip › PyDSTool/html/PyDSTool.Toolbox.optimizers.line_search.goldstein_rule.GoldsteinRule-class.html]

xml version="1.0" encoding="ascii"?


PyDSTool.Toolbox.optimizers.line\_search.goldstein\_rule.GoldsteinRule


| Home | Trees | Indices | Help | PyDSTool |
| --- | --- | --- | --- | --- |

Package PyDSTool ::
Package Toolbox ::
Package optimizers ::
Package line\_search ::
Module goldstein\_rule ::
Class GoldsteinRule

[hide private]

[frames] | no frames]

# Class GoldsteinRule

source code

```
object --+
         |
        GoldsteinRule
```

---

The Goldstein rule for a inexact line search


|  |  |
| --- | --- |
| Instance Methods | [hide private] |

|  |  |
| --- | --- |
| \_\_init\_\_(self, rho=0.1, alpha\_min=0.0, alpha\_max=1.0, \*\*kwargs)  Initializes the search Parameters : | source code |

|  |  |
| --- | --- |
| \_\_call\_\_(self, origin, function, state, \*\*kwargs)  Tries to find an acceptable candidate | source code |

**Inherited from `object`**:
`__delattr__`,
`__getattribute__`,
`__hash__`,
`__new__`,
`__reduce__`,
`__reduce_ex__`,
`__repr__`,
`__setattr__`,
`__str__`


|  |  |
| --- | --- |
| Properties | [hide private] |

**Inherited from `object`**:
`__class__`


|  |  |
| --- | --- |
| Method Details | [hide private] |

|  |  |
| --- | --- |
| \_\_init\_\_(self, rho=0.1, alpha\_min=0.0, alpha\_max=1.0, \*\*kwargs)  *(Constructor)* | source code |

Initializes the search Parameters :

- rho is the factor
- alpha\_min is the inf limit of the search interval (0.)
- alpha\_max is the max limit of the search interval (1.)

Those parameters should be tweaked depending on the function to
optimize

Overrides: object.\_\_init\_\_

  


| Home | Trees | Indices | Help | PyDSTool |
| --- | --- | --- | --- | --- |

|  |  |
| --- | --- |
| Generated by Epydoc 3.0.1 on Fri May 4 15:24:09 2012 | http://epydoc.sourceforge.net |
